# 

**Published:** 1897-08

**Authors:** 


### CONTENTS.

ORIGINAL COMMUNICATIONS—
General Considerations upon Major Anesthesia. By Robt. H. M. Dawbarn, M.D.. 361
Two Cases of Malignant Tumor Affecting the Breast, and their Treatment; With Some Remarks Upon Malignant Growths in General. By. M. B. Hutchins, M.D., Atlanta, Ga .......... 369
Blue Pyoktanin in the Treatment of Inoperable Malignant Growths. By Henry R. Slack, Ph.M., M D., LaGrange, Ga .......... 377
Report of a Successful Rhinoplastic Operation for Loss of the Nose. Also Operation for Removal of the Drum-Membrane and Necrosed Bones in Chronic Suppurative Aural Catarrh. By J. M. Crawford, M D., Atlanta, Ga .......... 384
Some Practical Thoughts Upon the Development of the Human Race and Obstetric Nursing. By R. R. Kime, M.D., Atlanta, Ga .......... 389

SOCIETY REPORTS—
Atlanta Society of Medicine .......... 397

CORRESPONDENCE—
The Plague in India .......... 402
Some Needed Legislation .......... 408

EDITORIAL—
The Committee on Legislation .......... 411
Osteopathy—What Is It? .......... 412
Examinations by State Board, Atlanta, June 29 .......... 413

SELECTIONS AND ABSTRACTS .......... 417
MEDICAL ITEMS .......... 428
BOOK REVIEWS .......... 431
MEDICAL ITEMS—Continued .......... x, xii, xiv
ADVERTISERS' INDEX TO ADVERTISEMENTS .......... xvi
CLASSIFIED INDEX TO ADVERTISEMENTS .......... xxxv

